# Rapidly Growing Huge Lower Back Malignant Peripheral Nerve Sheath Tumour

**DOI:** 10.7759/cureus.13712

**Published:** 2021-03-05

**Authors:** Naim Abuzarifa, Muath Mamdouh Mahmod Al-Chalabi, Wan Azman Wan Sulaiman

**Affiliations:** 1 Plastic and Reconstructive Surgery, Reconstructive Sciences Unit, Universiti Sains Malaysia, Pusat Pengajian Sains Perubatan Health Campus, Kota Bharu, MYS; 2 Surgery, Reconstructive Sciences Unit, Universiti Sains Malaysia, Pusat Pengajian Sains Perubatan Health Campus, Kota Bharu, MYS; 3 Plastic and Reconstructive Surgery, Universiti Sains Malaysia School of Medical Sciences, Kota Bharu, MYS

**Keywords:** malignant peripheral nerve sheath tumour, neurofibrosarcomas, neurofibromatosis

## Abstract

Malignant peripheral nerve sheath tumours (also called neurofibrosarcomas) are a rare, highly aggressive soft tissue sarcomas that arise from the peripheral nerves or cells associated with the nerve sheath, such as Schwann cells, peri-neural cells and fibroblasts. It is representing 10% of all soft tissue sarcomas in which it is considered as an extremely rare malignancy, especially in patients with neurofibromatosis type I. In the general population, it affects approximately 1 in 100,000 people. This article is reporting a 56-year-old Malay female patient who is a known case of neurofibromatosis type I for 20 years, presented with a lower back, pruritic, gradually increasing swelling during the last five months. Last month before the presentation, the lesion rapidly grows, reaching a size of (15×15 cm), accompanied by foul-smelling discharge and pain exacerbated with movement. Although no history of preceding trauma or accident, the mass bleeds within contact. In conclusion, only a few cases of giant malignant peripheral nerve sheath tumours reported in the literature describing its location and growth progression. We present a massive, extremely rapid growth of cutaneous exophytic malignant peripheral nerve sheath tumours over the lower back.

## Introduction

Malignant peripheral nerve sheath tumours are known as an aggressive and rare malignant soft tissues neoplasm [1-3]. It affects 1 in 100,000 people and approximately 3%-4.6% of patients with neurofibromatosis [4]. It arises from the peripheral nerve or cells related to the nerve sheath, such as Schwann cells and fibroblasts. Malignant peripheral nerve sheath tumours have a wide variety of histologic features and different reported names, including malignant neurilemmoma, neurofibrosarcoma, neurogenic sarcoma, and malignant schwannoma [5]. Usually, it developed from neurofibromas, especially in patients’ age groups between the second and sixth decades. Still, it can occur in any age group without gender preference [3,6]. Classically, the patient may present with a rapidly growing mass, associated with pain (sometimes radicular pain), paresthesia, and weakness. Malignant peripheral nerve sheath tumours can be found in any part of the body but often located in the neck, extremities, trunk or buttocks, and retroperitoneum. The prognosis may decrease in different circumstances, including the presence of neurofibromatosis, site and depth of tumour location, excisional margins, and tumour size. In patients with a tumour size >5 cm, it appears that tumour recurrence and metastases rate is higher [7].

This article reports a 56-year-old Malay female with underlying neurofibromatosis type I, presented with a rare giant cutaneous exophytic lower back malignant peripheral nerve sheath tumour measuring (15×15 cm) surgically excised.

## Case presentation

A 56-year-old Malay female patient is a known case of neurofibromatosis type I for 20 years, presented with a lower back, pruritic, gradually increasing swelling during the last five months. Last month before the presentation, the condition rapidly grew, accompanied by foul-smelling discharge and exacerbated pain with movement. Although no history of preceding trauma or accident, the mass bleeds within contact.

**Figure 1:**
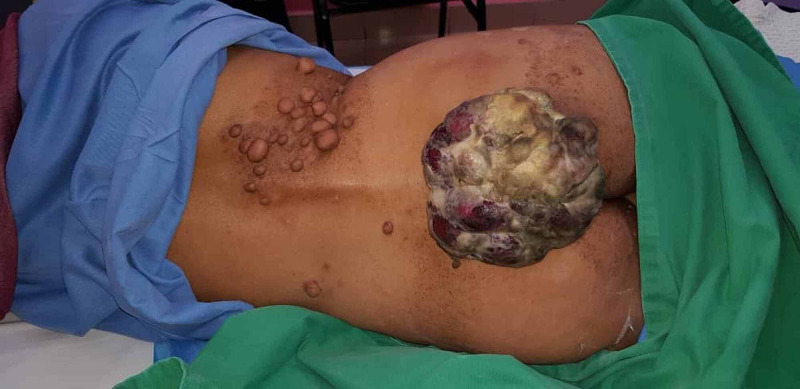
Huge lower back malignant nerve sheath tumour with neurofibromatosis type I.

Physical examination revealed a pedunculated, fungating, foul-smelling, and painful mass occupying the lower back. The range of movement in the hip is limited in all directions due to the mass's massive size, which measures approximately 15×15 cm, as shown in Figure 1.

**Figure 2:**
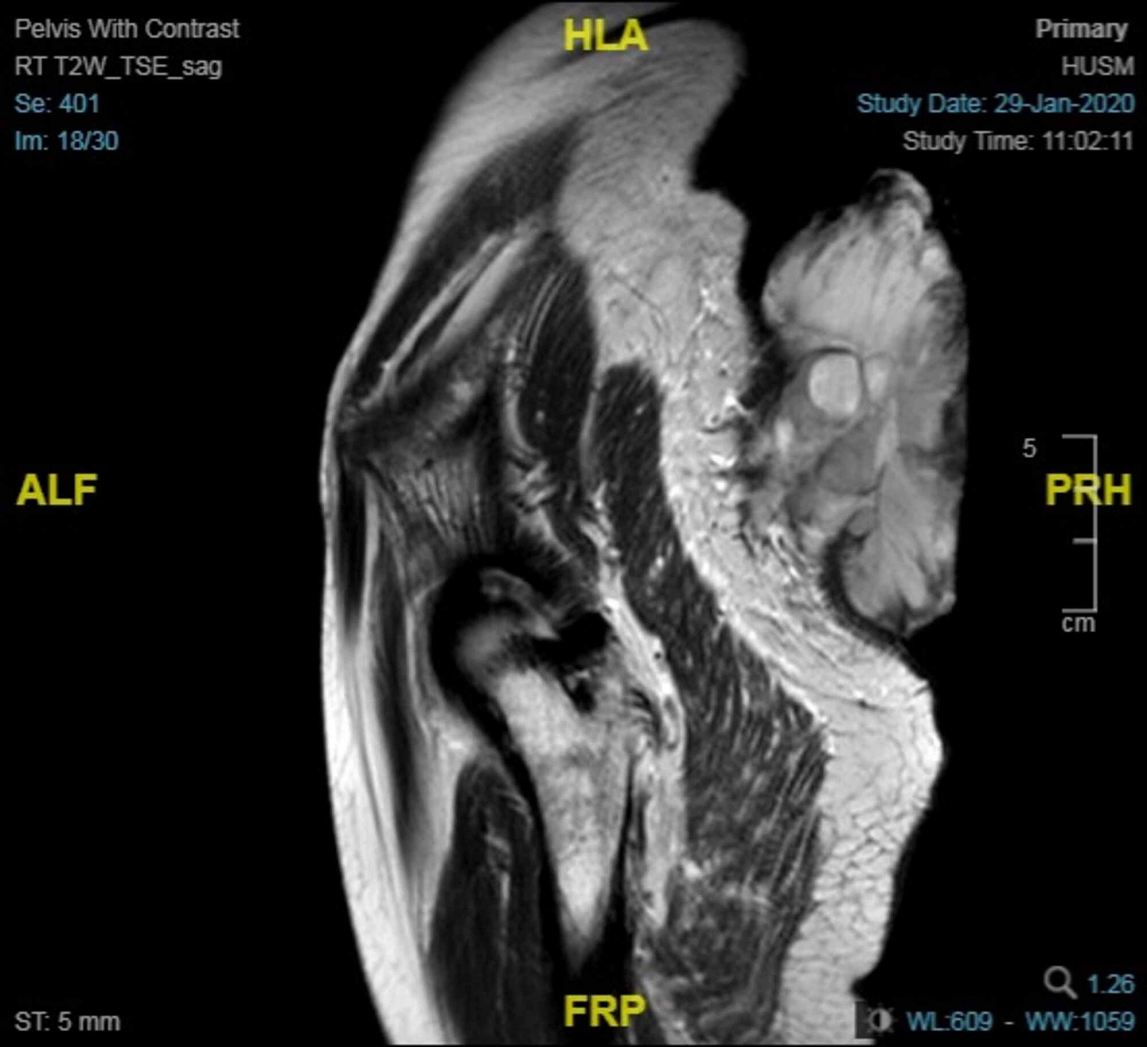
Longitudinal section MRI showing cutaneous pedunculated exophytic mass at the posterior pelvic region with subcutaneous fascia involvement.

Contrast MRI of the pelvis as in Figure 2 showed a cutaneous, pedunculated, exophytic lesion at the posterior pelvic region with the involvement of superficial subcutaneous fascia and intra-tumoral haemorrhage. No involvement of the deep fascia or underlying muscles.

**Figure 3:**
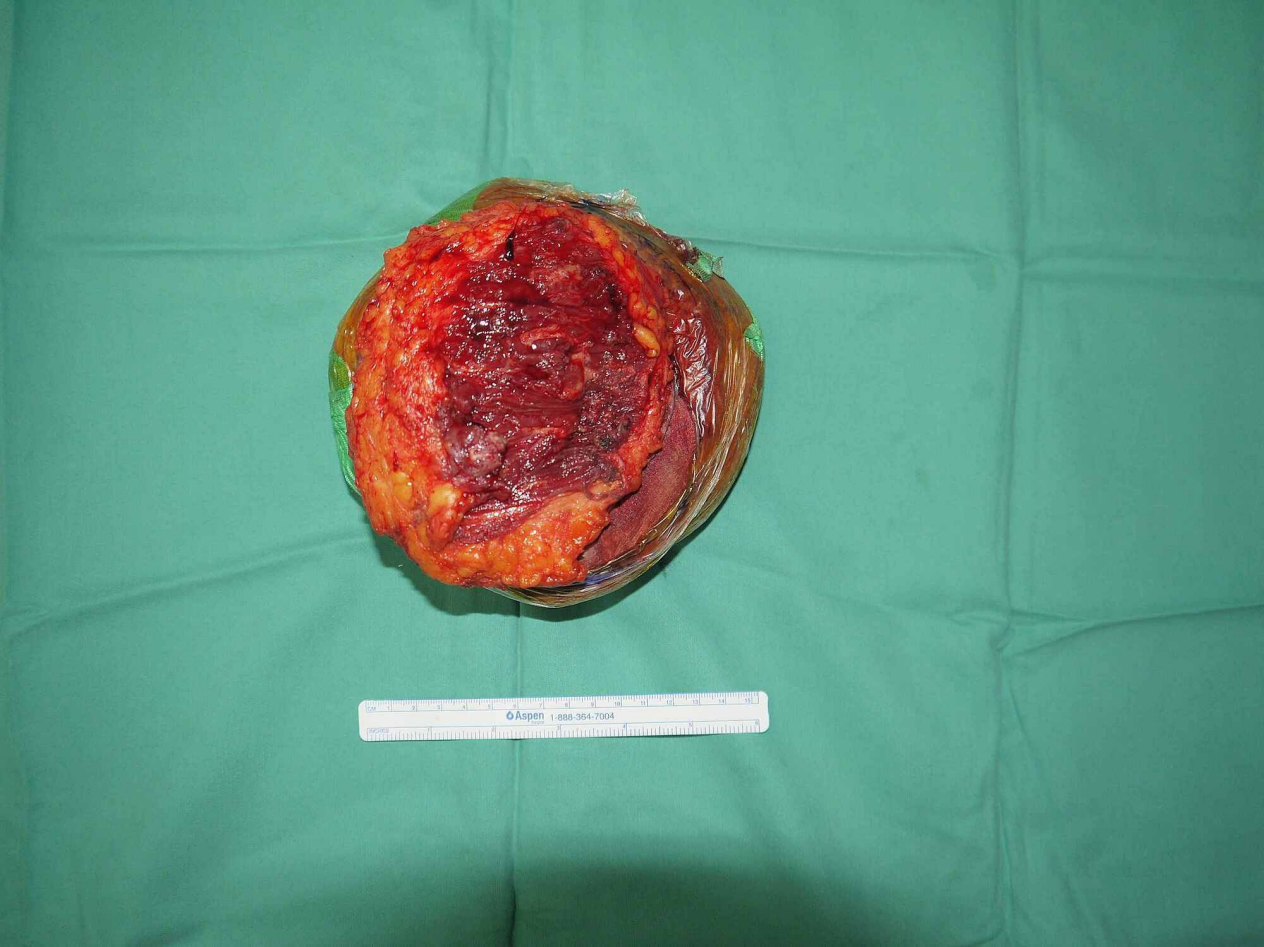
Excised tumour mass (15x15 cm).

Wide local surgical excision performed, the whole tumour removed (Figure 3) and defect reconstructed with rotational flap. Mass sent for histopathology, which reported malignant tumour cells composed of spindle-shaped cells embedded in densely cellular fascicles, alternate with hypo-cellular myxoid areas, confirming the diagnosis of malignant peripheral nerve sheath tumour.

## Discussion

Even with its rarity, the malignant peripheral nerve sheath tumour is a significant lesion because of its poor prognosis [5]. It is typically present as a rapid, aggressively growing painful lump. It accounts for approximately 10% of all soft tissue sarcomas [1-3,8]. It may develop suddenly in the general population, with an equal sex ratio [1,8,9]. 5% of cases are associated with neurofibromatosis type I. The most common age of diagnosis is ranging between the ages of 20 to 60 years. Regarding location, malignant peripheral nerve sheath tumour can occur in any part of the body, but usually in extremities (45%), trunk (35%), head and neck (20%), and retroperitoneal visceral area (15%) [1,3]. 

Hruban et al. [5] reported a study of 43 cases of malignant peripheral nerve sheath tumours located in the buttock and lower extremities; with a maximum dimension of the measure of the tumour 12.3 cm. On the other hand, Yoon et al. [1] reported a rare case of huge intrathoracic malignant peripheral nerve sheath tumour measuring (22×17×9 cm) in a 16-year-old girl with neurofibromatosis type I. Other reported case in the literature includes 51-year-old gentleman, reported by Abdel Al et al. [3] recurrent oval-shaped, large, exophytic, fungating, hemorrhagic, firm, and painful mass measuring (10×15 cm) on the right forearm. Fukushima et al. presented a 38-year-old Japanese man diagnosed as giant, ulcerated and hemorrhagic vertex malignant peripheral nerve sheath tumour with (21×19×9 cm) in dimensions. Moreover, two other reported cases by Panigrahi et al. [2] first case measures (15×7 cm) and located in the left orbit to the left temporal region, while second case measures (10×5 cm) founded over the left lumbar paraspinal area. Almost all these cases are slow growing over some time, with few cases of giant cutaneous exophytic malignant peripheral nerve sheath tumour. This article presents a case of aggressive, rapidly growing (over one month), and massive (15×15 cm) lower back cutaneous exophytic malignant peripheral nerve sheath tumour with underlying neurofibromatosis type I. 

Radiation therapy may be used to decrease the chance of the recurrence. In contrast, chemotherapy might be used if the whole tumour cannot be removed during surgery, or to treat the metastasis. Surgical excision still is the primary treatment for all patients. Up to 40% reported rates of local recurrence of malignant peripheral nerve sheath tumour occurs following surgical resection [10].

## Conclusions

Malignant peripheral nerve sheath tumour is one of the rare neoplasms, which can arise in any uncommon sites, other than its usual locations, and vary in the rate of growth. In this case report, we present a massive, extremely rapid growth of cutaneous exophytic malignant peripheral nerve sheath tumour over the lower back. 
